# *Wolbachia* affects mitochondrial population structure in two systems of closely related Palaearctic blue butterflies

**DOI:** 10.1038/s41598-021-82433-8

**Published:** 2021-02-04

**Authors:** Alena Sucháčková Bartoňová, Martin Konvička, Jana Marešová, Martin Wiemers, Nikolai Ignatev, Niklas Wahlberg, Thomas Schmitt, Zdeněk Faltýnek Fric

**Affiliations:** 1 Biology Centre CAS, Institute of Entomology, České Budějovice, Czech Republic; 2 Faculty of Science, University of South Bohemia, České Budějovice, Czech Republic; 3 Senckenberg German Entomological Institute, Müncheberg, Germany; 4 Department of Biology, Lund University, Lund, Sweden; 5 Faculty of Natural Sciences I, Institute of Biology, Zoology, Martin Luther University Halle-Wittenberg, Halle (Saale), Germany

**Keywords:** Population genetics, Entomology, Biogeography

## Abstract

The bacterium *Wolbachia* infects many insect species and spreads by diverse vertical and horizontal means. As co-inherited organisms, these bacteria often cause problems in mitochondrial phylogeny inference. The phylogenetic relationships of many closely related Palaearctic blue butterflies (Lepidoptera: Lycaenidae: Polyommatinae) are ambiguous. We considered the patterns of *Wolbachia* infection and mitochondrial diversity in two systems: *Aricia agestis*/*Aricia artaxerxes* and the *Pseudophilotes baton* species complex. We sampled butterflies across their distribution ranges and sequenced one butterfly mitochondrial gene and two *Wolbachia* genes. Both butterfly systems had uninfected and infected populations, and harboured several *Wolbachia* strains. *Wolbachia* was highly prevalent in *A. artaxerxes* and the host’s mitochondrial structure was shallow, in contrast to *A. agestis*. Similar bacterial alleles infected both *Aricia* species from nearby sites, pointing to a possible horizontal transfer. Mitochondrial history of the *P. baton* species complex mirrored its *Wolbachia* infection and not the taxonomical division. *Pseudophilotes baton* and *P. vicrama* formed a hybrid zone in Europe. *Wolbachia* could obscure mitochondrial history, but knowledge on the infection helps us to understand the observed patterns. Testing for *Wolbachia* should be routine in mitochondrial DNA studies.

## Introduction

*Wolbachia* is an intracellular bacterium massively infecting arthropods and filarial nematodes. As a maternally inherited symbiont, *Wolbachia* facilitates its spread by various methods of reproductive manipulation, such as cytoplasmic (sperm-egg) incompatibility, male killing, feminization, or parthenogenesis^1–3^. *Wolbachia* can be transferred also horizontally, using diverse vectors such as shared parasitoids, predators, or host plants^4–6^.

*Wolbachia* is passed to the next generation in a similar way as mitochondria. This fact causes selective sweeps in haplotypes due to genetic hitchhiking, followed by reduced mitochondrial diversity^7–9^. The subsequent effects are mito-nuclear discordance^10^ and deep divergences in mitochondrial phylogenies^11,12^. These may result in failures of mitochondrial DNA barcoding (i.e. using a short standardized DNA sequence) in the identification of species^1,13^.

Through these means*, Wolbachia* could interfere in diversification processes, but its role in arthropod evolution is still unclear. Speciation processes arise via the evolution of genetic barriers between populations^14^. When two different *Wolbachia* strains infect a host population, bidirectional cytoplasmic incompatibility may arise, leading to divergence^15^. On the other hand, when an infected and uninfected population meet, it can lead to a more common unidirectional cytoplasmic incompatibility. In such a case, the bacteria are predicted to spread faster than a gene flow barrier could evolve^16^. However, in theory, under specific circumstances of infection rate and migration among populations, even the unidirectional cytoplasmic incompatibility could lead to host genetic divergence^17,18^.

The existence of common horizontal transmission of *Wolbachia* between host species is supported by a general lack of congruence between the bacterial and host phylogenies, where either similar strains appear in phylogenetically distinct taxa, or closely related host taxa harbour distinct strains^19,20^.

Lycaenid butterflies (Lepidoptera: Lycaenidae) are known to be infected by the highest number of *Wolbachia* strains among butterfly families^21^. Within the lycaenids, Polyommatinae (the blues) often harbour unresolved phylogenetic relationships on lower taxonomical levels^22,23^, which may be further complicated by *Wolbachia* infection. *Wolbachia* may play an important role in butterfly mitochondrial structure, possible hybridization and even evolution. While its importance is becoming evident^24,25^, studies targeting patterns of *Wolbachia* infection across larger distribution areas are still scarce.

To shed some light on the complexity of infection by *Wolbachia* in Polyommatinae, we studied two systems of closely related blue butterflies, the widely distributed Palaearctic genera *Aricia* Reichenbach, 1817, and *Pseudophilotes* Beuret, 1958. The taxonomy of these systems relies on often subtle differences in morphology, mitochondrial DNA data, life histories, or habitat diversification^26–30^. The few nuclear markers classically used in butterfly phylogeny often fail to distinguish these species^28,29^. We focused on the taxa co-existing in Central Europe: *Aricia agestis* (Denis & Schiffermüller, 1775) and *A. artaxerxes* (Fabricius, 1793), as well as *Pseudophilotes baton* (Bergsträsser, 1779) and *P. vicrama* (Moore, 1865), and, for the latter genus, their closest relatives from other areas (i.e. the *Pseudophilotes baton* species complex).

We investigated the presence of *Wolbachia* infection in these butterflies using samples across their whole distributional ranges. We connected the *Wolbachia* infection patterns to the butterfly population structures inferred by a co-inherited marker, i.e. mitochondrial DNA. Our study will provide background information for future analyses of speciation, not only in the highly diverse group of Polyommatinae.

## Methods

### Study models

*Aricia agestis* and *A. artaxerxes* are cryptic species, which can be distinguished by mtDNA barcoding, but not by adult morphology in most of the area of their overlapping ranges^26,30^. Minor differences are in larval and pupal morphology^31^. *Aricia agestis* is a western Palaearctic species, inhabiting a wide range of mesic to xeric grasslands in lowlands of the Mediterranean and temperate regions. *Aricia artaxerxes* is a Palaearctic species reaching Northern Europe and inhabiting calcareous short-turf grasslands at higher elevations in the south. Their elevational ranges overlap in Central Europe^30^. They differ in the number of annual generations (multiple in *A. agestis*, single in *A. artaxerxes*). Host plants of both species are various Geraniaceae and *Helianthemum* Mill. spp. Several of their close relatives occur throughout Eurasia^28,32^, with west-Mediterranean *Aricia montensis* (Verity, 1928) being sister either to *A. artaxerxes*^28^ or *A. agestis*^33^.

*Pseudophilotes baton* and *P. vicrama* from the *P. baton* species complex are cryptic vicariant species: *P. baton* inhabits Western Europe (including the Alps and Italy) and *P. vicrama* occurs from Central Europe to Altai, western China and northern Mongolia. Both species are (or were in the recent past) present in Central Europe (Germany, Czech Republic, and Austria). They differ in the shape of male genitalia, which is used as a diagnostic trait^34^, but they share a similar mtDNA barcode in some cases^29^. Both inhabit xerothermic short turf grasslands, steppe-like sites in the case of *P. vicrama*, and pastures in mountains and their foothills in *P. baton*. Host plants are various species of *Thymus* L. There are several other taxa in the *Pseudophilotes baton* complex. *P. baton jacuticus* Korshunov et Viidalepp, 1980 inhabits steppe areas from the eastern and northern Baikal Lake surroundings to central and southern Yakutia, Russia. *Pseudophilotes panoptes* (Hübner, 1813) inhabits dry scrubby places in Iberia. *Pseudophilotes sinaicus* Nakamura, 1975 is present at a small area in the Sinai Peninsula Mountains (Egypt).

### Sampling and sequencing

To get sequences of mitochondrial DNA and examine the patterns of *Wolbachia* infection, we collected tissue samples of 115 individuals of *A. agestis*, 68 *Aricia artaxerxes*, 23 *Pseudophilotes baton*, 88 *P. vicrama* and 10 individuals of other *Pseudophilotes* (*P. sinaicus*: 4, *P. b. jacuticus*: 4, *P. panoptes*: 2) (Supplementary Dataset S1). In the case of *Pseudophilotes*, we dissected four male individuals from populations with mitochondrial sequences assigning them to a different species (Croatia, France), and 20 male individuals from all parts of the species complex’s distributional range to illustrate their diversity. The abdomens of male individuals were boiled for 5 min in 10% KOH to get the genital apparatus.

We extracted DNA with the Genomic DNA Mini Kit—Tissue (Geneaid) following the manufacturer’s protocols. To check the quality of extracted DNA and compare the mitochondrial structure with *Wolbachia* infection, we amplified, using polymerase chain reaction (PCR), the 5′ part of *cytochrome c oxidase subunit I* gene (COI), which is the most frequently used barcode in animals and hence a large reference library is available. We used the primer pair LCO/HCO and, when not successful, two pairs: LCO/K699 and Ron/HCO (primers:^35,36^). Only samples with a clear COI bands on agarose gels and obtained full COI sequence were included in the study.

We screened for the presence of *Wolbachia* in the host DNA samples by amplifying the *Wolbachia surface protein* gene (wsp) with the primer pair 81F and 691R^37^. Wsp is the common marker used to detect the presence of the bacteria (e.g.^38^). We included a negative control (PCR mixture with PCR H_2_O instead of DNA sample) and a positive control (a sample of *Pseudophilotes bavius* (Eversmann, 1832) with already sequenced wsp gene) in each PCR. To ascertain the presence or absence of *Wolbachia*, we ran 5 μl of the PCR product on 1.5% agarose gels. In the case of presence of a band ~ 700 bp long, the sample was scored as a positive and sequenced; otherwise it was scored as a negative. All samples were screened twice with wsp and sequenced once. In positive DNA samples, the *Wolbachia* gene *cytochrome c oxidase subunit I* (coxA) from *Wolbachia* multilocus sequence typing was amplified using primers coxA_F1 and coxA_R1^39^.

For all markers, we prepared PCR in 12.5 μl volume (6.25 μl Bioline 2 × MyTaq HS Red Mix, 4 μl PCR H2O, 0.625 + 0.625 μl primers; 1 μl DNA). The thermal cycling profile was 95 °C for 5 min; then 40 cycles of 94 °C for 30 s, 50 °C (COI)/55 °C (wsp, coxA) for 30 s, 72 °C for 90 s; and final extension 72 °C for 10 min. All the forward primers had T7 promoter and the reverse primers T3 universal tails attached.

We cleaned PCR products with enzymes FastAP and ExoI (Thermofisher) and sequenced them in one direction in Macrogen Inc. on ABI3730XL DNA analysers. We checked obtained sequences visually and aligned them in Geneious v. 8.0.5^40^. Wsp and coxA strains were defined using the reference sequences of the *Wolbachia* MLST database (^39^; https://pubmlst.org/wolbachia/). A possible double infection of the two arthropod *Wolbachia* supergroups in one individual^39^ may have been overlooked, but this should be rare within individuals (cf.^41^) and should not have any consequences on the conclusions of this study.

### Molecular data analyses

To ascertain the mitochondrial structure throughout the distribution ranges, in addition to our sequences, we used mitochondrial sequences of the target taxa and other species in these genera from BOLD^42^, publicly available in November 2019 (Supplementary Dataset S1). From the COI sequences collated in this study and in BOLD, we prepared three datasets: *Aricia agestis* (381 samples), *A. artaxerxes* (163), and *Pseudophilotes baton* complex (228) (Supplementary Dataset S1). While the barcodes of *Aricia agestis* and *A. artaxerxes* displayed nucleotide differences of about 2%^26^, the species *P. baton* and *P. vicrama*, together with the western Mediterranean *P. panoptes*, shared haplotypes^29^, and thus we treated them together as *P. baton* species complex.

To explore the butterfly population structure, we constructed the COI haplotype network by TCS statistical parsimony algorithm^43^ in POPART^44^. To cluster the *COI* sequences, we employed the hierarchical Bayesian Analysis of Population Structure (hierBAPS)^45^ in the package ‘rhierbaps’^46^ using R version 3.6.0^47^, without prior information on the geographic origin of each sample. To estimate the genetic diversity and to test the neutrality of the datasets (Tajima's D, Fu and Li's D* tests), we used DNASP v.5^48^.

To display the patterns of butterfly interpopulation genetic distances as genetic landscapes, we used the GDisPAL function of SPADS v. 1.0^49^ in R. First, we merged the sequences into 94, 43, and 69 populations (*A. agestis*, *A. artaxerxes* and *P. baton* complex, respectively) based on the geographic proximity of their localities (Supplementary Dataset S1). Second, we generated the genetic and geographic distance matrices in SPADS v. 1.0. The GDisPAL function is based on inverse distance-weighted interpolation (IDW)^50^. Here, the interpolation depends on distance values assigned at mid-points of each edge of a connectivity network built among the sampling localities (i.e., the Delaunay triangulation). Interpolation surfaces, visualised as heat maps, are affected by a distance weighting parameter *a*. We calculated the surface for *a* = 1–7. Considering potential correlation between genetic and geographic distances, we performed the distance interpolations using residual distances derived from the linear regression of genetic vs. geographic distances (inter-individual distances 1 of SPADS v. 1.0)^51^.

We utilized Bayesian analysis in Beast 1.10.4^52^ to unravel the mitochondrial genetic history in the two butterfly species groups and their closely related species. We transformed the samples of each species into haplotypes. Haplotypes shared among different taxonomic species entered the analysis in a number of copies corresponding to the number of these species. As outgroups, we used sequences of *Aricia anteros* (Freyer, 1838), *A. crassipuncta* (Christoph, 1893), and *A. morronensis* (Ribbe, 1910) in the *Aricia* tree, and sequences of *Pseudophilotes bavius* (Eversmann, 1832) and *P. fatma* (Oberthür, 1890) in the *Pseudophilotes* tree. This resulted in 118 samples in the *Aricia* and 71 samples in the *Pseudophilotes* input files. Beast analysis was run for 20 million MCMC generations, sampled every 5000 generations, for three independent runs. We employed the Coalescent Constant Size model with strict clock as the tree prior. Prior to the analysis, substitution models were selected in jModelTest^53^ by the lowest BIC, i.e. GTR + I + G for *Aricia* and GTR + G for *Pseudophilotes*. The outputs of the three runs were inspected in Tracer 1.6^54^. The first 10% of trees were discarded as burn-in. The trees from the three runs were combined and a single best ultrametric tree was obtained.

To explore the relationships among *Wolbachia* alleles, we used MrBayes 3.2.6^55^. We prepared four datasets with all sequences available: *Aricia* wsp (91 samples), *Aricia* coxA (81 samples), *Pseudophilotes* wsp (32 samples) and *Pseudophilotes* coxA (29 samples). As outgroups we used sequences of *Wolbachia* extracted from the nematode *Litomosoides sigmodontis* (Chandler, 1931) (GenBank codes: wsp: AF409112, coxA: FJ390246). Substitution models were GTR + G in *Aricia* wsp, and HKY in *Pseudophilotes* wsp, *Aricia* coxA and *Pseudophilotes* coxA. MrBayes was run for 20 million MCMC generations, sampled every 5000 generations, with temperature = 0.2, four simultaneous chains, four independent runs. The first 10% of trees were discarded as burn-in. We inspected the convergence of the four runs and the sufficient sampling by the *sump* command based on the log likelihood and high effective sample sizes. The trees from the four runs were summarized under the 50% majority-rule consensus. We inferred Bayesian trees in the CIPRES Science Gateway^56^.

## Results

### *Aricia*: butterfly mitochondrial structure

**Figure 1:**
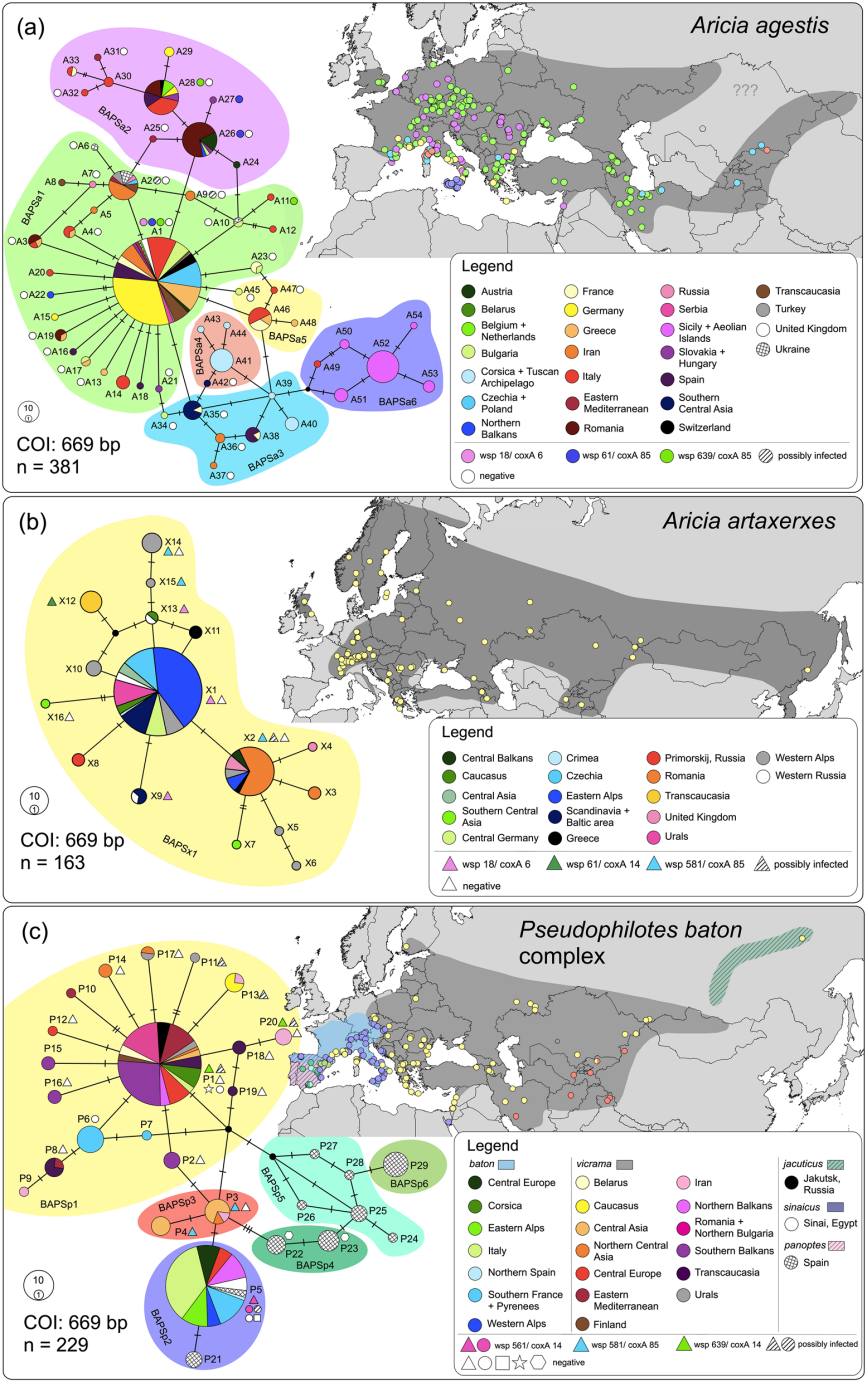
TCS haplotype networks of (**a**) *Aricia agestis*, (**b**) *Aricia artaxerxes* and (**c**) *Pseudophilotes baton* species complex, based on COI. The colours of map points correspond to BAPS clusters (coloured network background). The shades on the maps represent the distribution of each taxon. The specimens in the networks were allocated to geographical or political regions, for readers’ orientation. The haplotypes, in which some of the individuals were inspected for *Wolbachia* infection, are indicated, with a different mark for each bacterial strain. These marks correspond to Fig. 3; and in (**c**), circle = *P. baton,* triangle = *P. vicrama*, square = *P. sinaicus*, star = *P. b. jacuticus*, hexagon = *P. panoptes.* Note that COI sequences not marked with any symbol were obtained in databases and hence not tested for *Wolbachia*. Maps were created in QGIS v. 2.18 (http://qgis.org) and the graphics was compiled in Graphic for Mac v. 3.1 (https://www.graphic.com/mac/).

**Figure 2:**
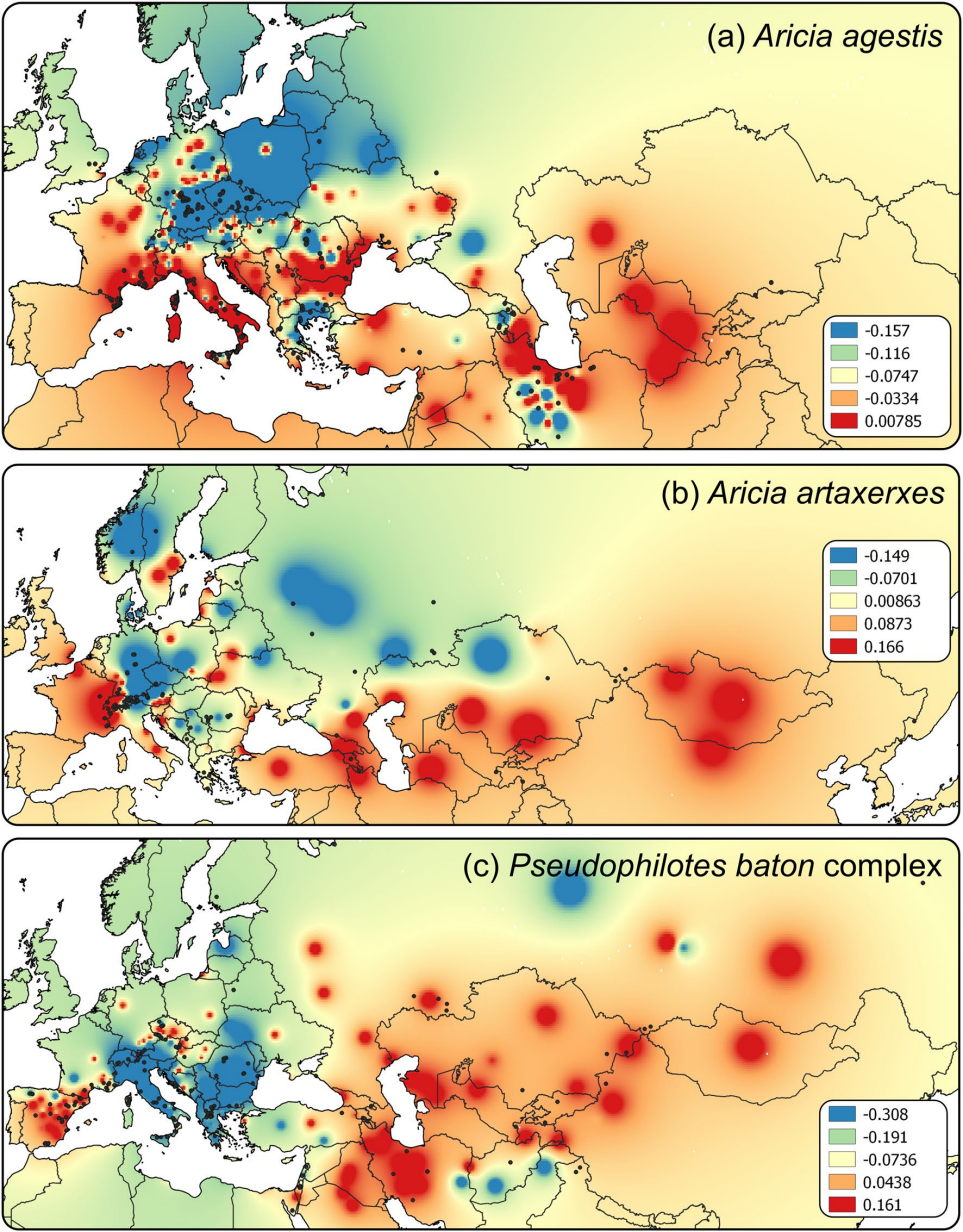
Genetic landscapes based on COI genetic distances among individual populations of (**a**) *Aricia agestis*, (**b**) *Aricia artaxerxes* and (**c**) *Pseudophilotes baton* species complex, created by the GDisPAL function in SPADS. The distance weighting parameter *a* = 3 was used as a presentable visualization. The map colours represent residuals of genetic distances. Maps were created in QGIS v. 2.18 (http://qgis.org) and the graphics was compiled in Inkscape v. 1.0 (https://inkscape.org/).

In the dataset of 381 sequences of *Aricia agestis*, the TCS analysis revealed 54 haplotypes (A1–A54, 35 segregating sites) belonging to six BAPS clusters (Fig. 1a). The first cluster BAPSa1 harboured the majority of all samples, showing a star-like topology typical for recent population expansions; this cluster was found all over the distribution range of the species, except for Central Asia. The second cluster BAPSa2 was restricted to Western, Central and Southern Europe plus one sample from the Levant. The clusters BAPSa3 and BAPSa5 were found in the Mediterranean fringe of Europe, but both were relatively rare; in addition, BAPSa3 was also found from Alborz (northern Iran) to Central Asia. The BAPSa4 cluster was mostly restricted to Corsica and adjoining islands, plus one sample from Alatau (Central Asia). The BAPSa6 cluster was obtained for Sicily, the Aeolian Islands and one sample from the mainland side of the strait of Messina (haplotype A49). This genetic structure was mirrored by the genetic landscape analysis (Fig. 2a), showing the highest genetic distances along the Mediterranean fringe of Europe from southern France to the northern Balkans. The dataset’s nucleotide diversity was 0.0044, Tajima's D was not significant (− 1.634; p > 0.05), Fu and Li's D* test was significant (− 3.020; p < 0.05). The latter test suggested the excess of singleton haplotypes.

In the dataset of 163 sequences *Aricia artaxerxes*, the TCS analysis revealed 16 haplotypes (X1–X16, 15 segregating sites) belonging to a single BAPSx1 cluster (Fig. 1b). The genetic landscape showed the highest distances among the samples from the Western Alps, in the Caucasus and in Central Asia (Fig. 2b). A barrier appeared also between south-eastern and Central Europe. The dataset’s nucleotide diversity was 0.0019, which is 2.3 times lower than in *A. agestis*. Neither Tajima's D (− 1.706; p > 0.05) nor Fu and Li's D* (− 1.796; p > 0.1) tests were significant.

In the mitochondrial Beast tree, haplotypes of *A. agestis* and *A. artaxerxes* formed well-defined monophyletic groups. The closest relative of *Aricia artaxerxes* was the Iberian *A. montensis*, with *A. agestis* being sister to this clade (Supplementary Fig. S1). Within *A. agestis*, Sicilian haplotypes (cluster BAPSa6) formed a well-supported lineage. A part of the haplotypes from cluster BAPSa2 (A28-A33) formed another separate lineage. Within *A. artaxerxes*, samples from Kyrgyzstan formed a well-supported clade probably representing a distinct lineage within the species (^32^; these samples were not used in further analyses).

### *Aricia*:* Wolbachia*

**Figure 3:**
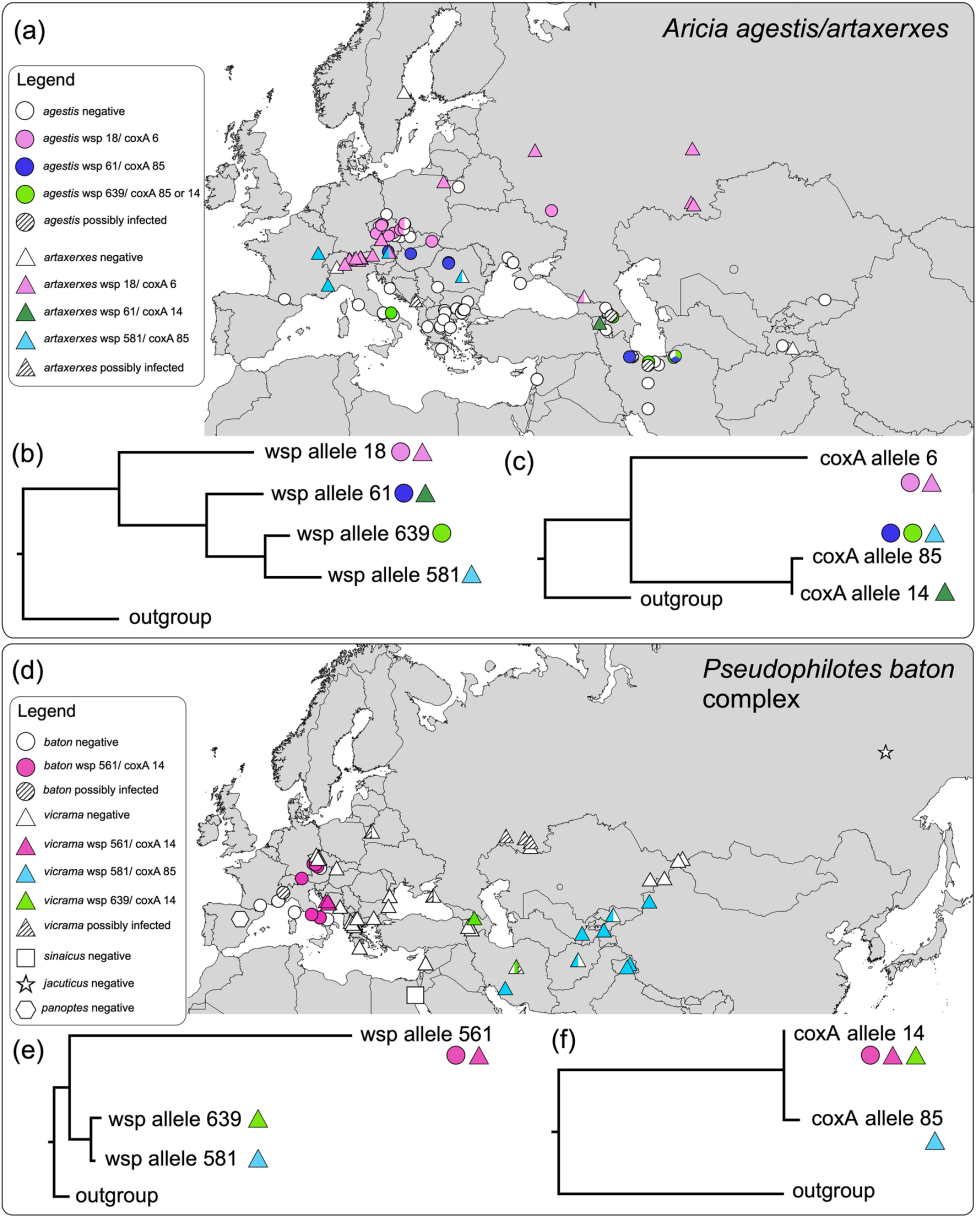
*Wolbachia* infection in two groups of closely related lycaenid butterflies: (**a**–**c**) *Aricia agestis*/*A. artaxerxes*: (**a**) distribution map, (**b**) Bayesian phylogenetic tree (MrBayes) of wsp, and (**c**) of coxA alleles. d–f *Pseudophilotes baton* species complex, (**d**) distribution map, (**e**) Bayesian phylogenetic tree of wsp, and (**f**) of coxA alleles. For a part of the samples, only the wsp gene was obtained, such samples were categorized by their wsp alleles. Maps were created in QGIS v. 2.18 (http://qgis.org) and the graphics was compiled in Graphic for Mac v. 3.1 (https://www.graphic.com/mac/).

Our analyses revealed that 29 *Aricia agestis* individuals out of 115 tested were positive for *Wolbachia* (prevalence: 22%). Of these, 27 were successfully sequenced for wsp and 17 for coxA. All positive host samples were from the widely distributed groups BAPSa1 and BAPSa2. Sequencing revealed three wsp alleles and three coxA alleles, all already known (Table 1). The wsp allele 61 was congruent with coxA allele 85, wsp allele 639 accorded to coxA alleles 85 and 14, and wsp allele 18 to coxA allele 6. Two different wsp alleles were discovered in the same locality in Iran. Three different wsp alleles and three coxA alleles were discovered in the COI butterfly haplotype A1 (Table 1). However, the *Wolbachia* alleles were separated geographically in this case (Fig. 3a): Wsp allele 18/coxA allele 6 (together referred as a strain) were distributed northerly of the Alps and wsp alleles 61 and 639/coxA 85 were distributed southeast of the Alps.

**Table 1:** *Wolbachia* wsp and coxA alleles detected in samples of *Aricia* spp. and *Pseudophilotes* spp. Country codes accord to ISO-3 standard; W RUS = western Russia, RUS: Cauc = Russia, Caucasus Mts. In *A. agestis* wsp 639, two samples were recognized as coxA 14 (host haplotype A1, from eastern Iran), one as coxA 85 (host haplotype A1, from Georgia), and in four cases, the sequencing of coxA was not successful.

Species	wsp allele	coxA allele	Host haplotype	Host mtDNA BAPS cluster	Prevalence in host haplotypes	Geographical distribution (positive samples)
*A. agestis*	18	6	A1	BAPSa1	11/64	CZE + W RUS + SVK
*A. agestis*	61	85	A1, A26, A27	BAPSa1, BAPSa2	2/64, 5/7, 1/1	IRN, ROU + AUT, HUN
*A. agestis*	639	85 and 14	A28, A1, A11	BAPSa2, BAPSa1	1/3, 6/64, 1/1	ITA, GEO + IRN, IRN
*A. artaxerxes*	18	6	X1, X9, X13	BAPSx1	49/51, 1/1, 1/1	CZE + RUS + POL + AUT + ITA + CHE, W RUS, RUS: Cauc
*A. artaxerxes*	581	85	X2, X14, X15	BAPSx1	5/7, 1/2, 1/1	ROU + AUT, FRA, FRA
*A. artaxerxes*	61	14	X12	BAPSx1	5/5	ARM
*P. baton*	561	14	P5	BAPSp2	11/13 in *P. baton* P5	CZE + ITA + AUT
*P. vicrama*	561	14	P5	BAPSp2	7/7 in *P. vicrama* P5	HRV
*P. vicrama*	581	85	P3, P4	BAPSp3	5/6, 4/4	IRN + AFG + CHN + IND, TJK + KGZ
*P. vicrama*	639	14	P1, P20	BAPSp1	2/41, 2/3	GEO, IRN

In *A. artaxerxes*, 64 out of 69 tested individuals were positive for *Wolbachia* (prevalence: 93%); 63 were successfully sequenced for wsp, 62 for coxA. The sequencing revealed three wsp alleles and three coxA alleles, all already known. Wsp allele 18 (and sequences with 1–2 differing bases) corresponded with coxA allele 6, wsp allele 581 with coxA allele 85, and wsp allele 61 with coxA allele 14. Alleles were distributed geographically (Fig. 3a). The strain wsp 18/coxA 6 was distributed north of the Alps (similarly as in *A. agestis*) where it met with wsp 581/coxA 85 in the Alps. The strain wsp 61/coxA 14 was only detected in Transcaucasia.

In the *Wolbachia* wsp tree (Fig. 3b, Supplementary Fig. S1), the widely distributed wsp allele 18 split first, containing samples of the bacteria from *A. artaxerxes* across Europe and several samples from *A. agestis* from the Czech Republic, Slovakia and western Russia. The rest of the samples formed a monophyletic branch, within which (1) *Wolbachia* from *Aricia agestis* from Europe and Iran formed a clade together with that from Armenian *A. artaxerxes* (allele 61), (2) material from *Aricia agestis* from Iran, Georgia and one individual from Italy formed a second clade (allele 639), sister to (3) the third clade, detected in *A. artaxerxes* from France, Romania, and Austria (allele 581). The *Wolbachia* coxA tree (Fig. 3c) split into two branches: (1) the widely distributed allele 6 found in both *A. agestis* and *A. artaxerxes*, and (2) the closely related alleles 14 and 85, found in samples of both butterfly species from Iran and Transcaucasia, but also from Eastern Europe and the Alps.

### *Pseudophilotes*: butterfly mitochondrial structure

In the 228 sequences of *Pseudophilotes* spp., the TCS analysis revealed 29 haplotypes (P1–P29, 23 segregating sites) belonging to six BAPS clusters (Fig. 1c). In several cases, the assignment to a BAPS cluster did not correspond to the taxonomical species revealed by genitalia dissection: BAPSp1 (P1–2, P6–20) contained mostly *P. vicrama* from Europe, but also the north-Asian *P. b. jacuticus* and *P. baton* from France, Corsica, and eastern Italy (the latter with “*baton*” valva type, Supplementary Fig. S2); this cluster had a star-like topology typical for recent population expansions. BAPSp2 (P5, P21) contained mostly *P. baton*, but also *P. vicrama* from adjacent distributional regions of Austria and Croatia (“*vicrama*” valva type: Supplementary Fig. S2 and ABOLD478-16), *P. sinaicus* and *P. panoptes*. BAPSp3 (P3–4) included samples of *P. vicrama* (“*vicrama*” valva type) from Central Asia (Afghanistan, westernmost China, northernmost India, Iran, Kyrgyzstan, Tajikistan). BAPSp4–6 harboured only samples of *P. panoptes*. The dataset’s nucleotide diversity was 0.0046. Neither Tajima's D (− 1.106; p > 0.1) nor Fu and Li's D* (− 1.729; p > 0.1) tests were significant. The genetic landscape (Fig. 2c) showed the highest differentiation in western Iran and Central Asia and then in the Iberian Peninsula and southern France. The distribution of the mitochondrial species delimitation is deviating from the one based on male genital structures with the first running a bit more to the east than the second, i.e. crossing the Czech Republic, running through the border regions between eastern Austria and Slovakia, as well as between Croatia and Bosnia and Herzegovina.

**Figure 4:**
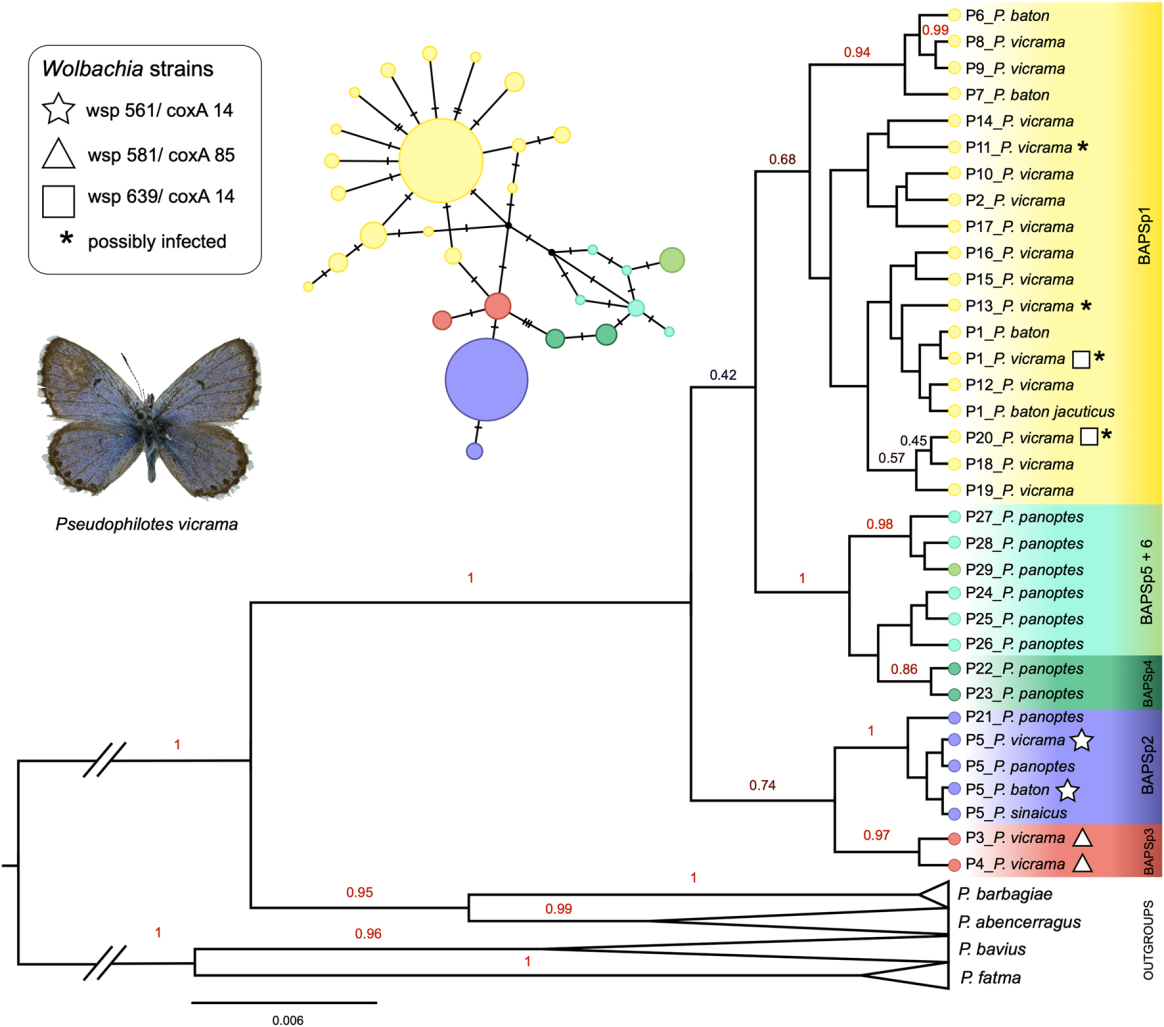
Mitochondrial Bayesian phylogenetic tree of *Pseudophilotes* spp. COI haplotypes. The branch labels represent posterior probabilities > 0.4. Haplotypes positive for *Wolbachia* are marked. The tree was visualized in FigTree v. 1.3.1 (https://github.com/rambaut/figtree). Graphics was compiled in Graphic for Mac v. 3.1 (https://www.graphic.com/mac).

The Beast tree (Fig. 4) revealed two clades: the first clade contained two lineages: BAPSp3 (P3–4, *P. vicrama* from Central Asia) and BAPSp2 (*P. baton, P. sinaicus*, part of *P. panoptes, P. vicrama* from the contact zone). The second clade split into two lineages: the first one consisted of *P. panoptes* haplotypes (except for those in BAPSp2) and the second of *P. vicrama* from BAPSp1, *P. b. jacuticus* and *P. baton* from southern France.

### *Pseudophilotes*:* Wolbachia*

Looking for *Wolbachia* presence, 37 out of 108 tested individuals were positive (prevalence: 34%): 12 out of 17 in *P. baton* (71%), 25 out of 81 in *P. vicrama* (31%), 0 out of 4 in *P. sinaicus* (0%), 0 out of 4 in *P. b. jacuticus* (0%), and 0 out of 2 in *P. panoptes* (0%)*.* The samples positive for *Wolbachia* followed geographic structures (Fig. 3d): 9 out of 36 in BAPSp1 (25%, positive samples from Iran, Georgia, western Russia and Belarus, but none from Western, Central and south-eastern Europe), 19 out of 24 in BAPSp2 (79%, Central Europe, Italy, Croatia; the majority of negative samples were *P. sinaicus*), 9 out of 10 in BAPSp3 (90%, Central Asia; one negative sample from Afghanistan), and 0 out of 2 in BAPSp4. BAPSp5 and BAPSp6 were based on database samples only and thus the *Wolbachia* presence could not be tested. Thirty-one samples were successfully sequenced for wsp, and 28 for coxA. We revealed three wsp alleles and two coxA alleles, all previously known. Wsp allele 561 corresponded with coxA allele 14 and was restricted to BAPSp2 (haplotype P5; *P. baton* and *P. vicrama* from the contact zone). Wsp allele 639 also accorded to coxA allele 14 and this combination was found in two Georgian and one Iranian samples (P1, BAPSp1). Wsp allele 581 in combination with coxA allele 85 was only obtained for Central Asia (BAPSp3, haplotypes P3–4).

The *Wolbachia* wsp tree (Fig. 3e, Supplementary Fig. S1) showed that wsp allele 561 present in BAPSp2 (*P. baton* and *P. vicrama* from the contact zone) formed a separate lineage from the closely related alleles 639 and 581 (Central Asian *P. vicrama* from BAPSp3 and three samples from Georgia and Iran from BAPSp1). The two coxA alleles (Fig. 3f, Supplementary Fig. S1) differed in a single mutation. *Pseudophilotes vicrama* from Georgia hence harbour *Wolbachia* related to those from Central Asian *P. vicrama* (BAPSp3) in wsp and those from *P. baton* (BAPSp2) in coxA.

## Discussion

Two closely related groups of Polyommatinae butterflies displayed a composite pattern of *Wolbachia* infection, containing both infected and uninfected populations and several different *Wolbachia* strains, which were linked both to their mitochondrial history and to their geographical distribution.

### *Wolbachia* infection in *Aricia agestis* and *A. artaxerxes*

*Wolbachia* was highly prevalent in *Aricia artaxerxes* throughout its whole distribution range, in contrast to *A. agestis*. *Aricia agestis* and *A. artaxerxes* are believed to represent well-defined species. In the COI barcode, there is a constant minimum p-distance of about 2% between them, sufficient to distinguish two species^26,28^. They are not distinguished by some nuclear markers (e.g. internal transcribed spacer 2^28^) but are well separated by others as allozymes^57^. Lineages distinguished by mtDNA and allozymes also differ in voltinism. A possibly analogous situation was observed in a cryptic species pair of Hesperidae butterflies, where *Spialia rosae* Hernández-Roldán, Dapporto, Dincă, Vicente & Vila, 2016 differed from *S. sertorius* (Hoffmannsegg, 1804) by the COI barcode, specific cuticular hydrocarbons, their host plants, and the presence of *Wolbachia*^58^. *Wolbachia* presence thus might serve as a further distinguishing trait of the cryptic species pair *A. agestis* and *A. artaxerxes*.

The mitochondrial diversity of the highly infected *A. artaxerxes* was low compared to much less affected *A. agestis* (16 vs. 54 haplotypes, respectively). This represents indirect evidence for a selective sweep in *A. artaxerxes*. Such a probable selective sweep was also observed in the Chinese hesperid butterfly *Polytremis nascens* Leech, 1893, with *Wolbachia* infected populations being genetically less diverse than uninfected ones^59^.

### Overlapping areas of *Aricia agestis* and *A. artaxerxes* and transfer of *Wolbachia*

A proportion of the examined *Aricia agestis* individuals were also positive for *Wolbachia.* In both *Aricia* species, we discovered several *Wolbachia* strains. Those samples of *A. agestis* from lowland Central and north-eastern Europe, which were positive for *Wolbachia*, were infected by the wsp 18/coxA 6 strain, also found in *A. artaxerxes* in this region (Fig. 3a). At Hochschwab, a mountain massif within the north-eastern limestone Alps (Austria), the barcodes of both species co-occur, infected by a similar coxA allele 85 and two wsp alleles (581 and 61).

Although *Aricia artaxerxes* inhabits higher elevations in Southern Europe than *A. agestis*, their elevational distribution overlaps in Central and Eastern Europe^26,30^. Our specific Hochschwab locality (Seebergsattel pass) is situated at 1260 m a.s.l., and a sequence of an *A. agestis* specimen from an even higher elevation (about 1600 m a.s.l.), 30 km away from our record, is published (Hochkar, Austria: ABOLD655-17).

The occurrence of similar *Wolbachia* alleles found in the two *Aricia* species in lowland Central Europe suggests a transfer of this bacterium between these species. Hybridization of the two species was documented alongside a contact zone established during Holocene in Great Britain^60^. However, sharing the same *Wolbachia* allele between the two butterfly species is unlikely a product of hybridization, as the host’s mitochondrial DNA would be transferred together with *Wolbachia*. In this context, Sintupachee et al*.*^5^ observed phylogenetically distant insect groups infected by the same *Wolbachia* strain utilizing a similar host plant, suggesting a horizontal transfer. Thus, hemipteran insects, injecting their saliva containing bacteria to plant cells, could have infected leaf-chewing insects via this means. Larvae of leaf-mining lepidopterans (Gracillariidae) were also found to introduce *Wolbachia* into plant tissue, and evidence of horizontal transfers was detected in the comparison of their phylogeny and *Wolbachia* infection^61^. Larvae of *Aricia agestis* and *A. artaxerxes* share their host plants, which may have served as vector for the infection. Alternatively, *Wolbachia* might be transferred horizontally through a shared parasitoid^62^.

Situation in Hochschwab, where a population contained the barcodes of both species, and two different wsp alleles observed also in other parts of their ranges, could be product of a past hybridization and would require further attention.

### *Aricia agestis* and *A. artaxerxes* biogeographic history

The two examined *Aricia* species strongly differ in their biogeographic patterns. On the one hand, *Aricia artaxerxes* is a boreo-montane species, with decreased mitochondrial diversity and thus with its mitochondrial history obscured by *Wolbachia* infection. However, *Wolbachia* infection could also give us some hints to the butterfly’s phylogeographic history. The different *Wolbachia* strains associated with spatially separated *A. artaxerxes* haplotypes suggest the existence of two host haplogroups (not distinguished in BAPS analysis): (1) a Central European group reaching Northern Europe and the Russian forest-steppe belt (infected by *Wolbachia* wsp allele 18/coxA allele 6, host haplotypes X1, X9, X13), and (2) a south-western European group (*Wolbachia* wsp allele 581 + two closely related coxA alleles, host haplotypes X2, X15, X14). Both groups are present in the Alps and in the Balkan Peninsula. The current boreo-montane range with such longitudinally distributed haplogroups could be a legacy of a widespread distribution in the Pleistocene mammoth steppe biome, with remnants of such distribution existing in the Holocene^63^, the result of postglacial expansion from refugia located north of the Mediterranean region^64^, or a mixture of both.

*Aricia agestis*, on the other hand, is a temperate grassland species with the highest genetic diversity in the Italian and the Balkan peninsulas (Fig. 2b). The observed genetic patterns call for glacial retreats in peninsular Italy and the Balkan Peninsula as often observed in warm-adapted species in Europe^65,66^, with both expanding from these retreats postglacially^67^. During this postglacial expansion through Europe, the populations stemming from both refugia were mixing in a low density hybridisation system (cf.^66^) and, in addition, occasionally hybridize with *A. artaxerxes*^60^ and also might have been infected by *Wolbachia* from this species. The mitochondrial clusters BAPSa4 and BAPSa6 restricted to Corsica and adjoining islands, as well as Sicily, respectively, indicated further independent differentiation centres on these two islands due to their rear edge position^68^. Whereas the biogeographically isolated position of Corsica (and Sardinia) has been known for some time^65^, Sicily, separated by just 3 km of sea from the southernmost tip of peninsular Italy, has been recovered as being biogeographically particular only recently^69–71^. In addition, the isolated occurrence of the eastern branch BAPSa3 from north-east Iran to Central Asia calls for a separate retreat in this area; however, with its glacial-interglacial dynamics still being ambiguous.

### *Wolbachia* distribution in the *Pseudophilotes baton* species complex

The patterns of *Wolbachia* infection in the *Pseudophilotes baton* species complex correspond to the mitochondrial history of the butterflies, which does not agree with the described taxa (Figs. 1c, 3d). The phylogenetics of closely related *Pseudophilotes* species from the *Pseudophilotes baton* complex is traditionally problematic. These species were not distinguished sufficiently by four nuclear genes^29^. *Pseudophilotes vicrama* is the most widely distributed member of the complex, present from Central Europe to Mongolia, distinguished by the shape of its valvae from the putative taxa occurring at the margins of its distribution, i.e. *P. baton, P. b. jacuticus*, *P. sinaicus*, and *P. panoptes*. The COI barcodes of the butterflies do not correspond to this taxonomic classification. Thus, *P. b. jacuticus* shares haplotype P1 with *P. vicrama* from Europe; and *P. sinaicus* shares haplotype P5 with the majority of *P. baton*. With the data available, it therefore seems impossible to decide whether the complex represents completely separated species with secondary contacts, a speciation process under way, or just a single species with polymorphic male genitalia (cf. bad species concept^72^).

Similarly to the situation in *Aricia agestis* and *A. artaxerxes*, the *Pseudophilotes* mitochondrial clusters with high *Wolbachia* prevalence rates (BAPSp2, BAPSp3) harboured considerably lower genetic diversity (two haplotypes each) than the mostly uninfected BAPSp1 cluster (17 haplotypes), again pointing to a selective sweep in the heavily infected clusters.

Three different *Wolbachia* strains were detected in this butterfly species complex, found in different parts of its distribution area (Fig. 3d). *Wolbachia* was highly prevalent in *P. baton* (host mtDNA cluster BAPSp2), except for the samples from southern France, Corsica, and two samples from Italy, which were negative in *Wolbachia* and clustered with *P. vicrama* in the host mtDNA. Another *Wolbachia* strain was found in *P. vicrama* from Central Asia, which mtDNA also formed a separate cluster (BAPSp3). The cluster BAPSp1 was mostly uninfected, except for some samples from western Russia (samples from which we failed to obtain *Wolbachia* sequences, Supplementary Dataset S1) and samples from Iran and Georgia. These latter were infected by a strain related to the one found in BAPSp3 in wsp, and to the one in BAPSp2 in coxA (Fig. 3d).

In the host mitochondrial Bayesian tree, the two clusters infected by *Wolbachia*, BAPSp3 (Central Asian *P. vicrama*) and BAPSp2 (mostly *P. baton*), formed a clade separated from the mostly uninfected samples. However, BAPSp2 and BAPSp3 were infected by two different *Wolbachia* strains. As these two strains are genetically differentiated from each other and geographically located on opposite angles of the range (i.e. south-central Europe vs. Central Asia), two independent infections with similar consequences is a likely scenario.

All in all, the *Pseudophilotes* species complex follows the butterfly paradigm of glacial refugia and postglacial range dynamics in Europe^73^ with a non-expansive taxon in Iberia (i.e. *P. panoptes*) and expansive lineages out of the Adriatic- (i.e. *P. baton*) and the Pontic-Mediterranean (i.e. *P. vicrama*) refugia^67^. Similar to *A. agestis*, *P. vicrama* seems to be polycentric with at least one western centre of differentiation (i.e. BAPSp1) from the Balkans to Iran (i.e. Pontic-Mediterranean-Iranian elements, cf.^74^) and one Central Asian element (BAPSp3) with still unknown range dynamics, also supported by a *Wolbachia* type restricted in *P. vicrama* to this genetic lineage. Additionally, *P. b. jacuticus* might represent a Siberian element with a Siberian centre of dispersal (cf.^65^), while *P. sinaicus* might be a rear edge population with in situ differentiation and adaptation to the prevailing eremic conditions.

### Hybrid zone between *Pseudophilotes baton* and *P. vicrama*

Sequences of *Pseudophilotes vicrama* from Croatia and lowland eastern Austria are assigned to haplotype P5 (BAPSp2), mostly found in *P. baton*; furthermore, the Croatian samples also harboured the same *Wolbachia* strain as *P. baton*. However, their genital structures correspond to the “*vicrama*” type (Supplementary Fig. S2). In addition, two samples of *P. baton* from south-eastern Italy were assigned as the *P. vicrama* haplotype P1 and were negative for *Wolbachia*. This implies the existence of a hybrid zone between these two taxa crossing Central Europe. This region is a known contact zone between eastern and western lineages of many invertebrates and vertebrates, e.g., in the lycaenid butterfly *Polyommatus coridon*^75^, mice *Mus musculus* and *M. domesticus*^76^, and toads *Bombina bombina* and *B. variegata*^77^.

*Pseudophilotes baton* samples from southern France and Corsica also lacked *Wolbachia* and were assigned to the BAPSp1 cluster, otherwise confined to *P. vicrama*, suggesting that hybridization and population replacement might be multiple within the species complex. Furthermore, the distribution of this mitochondrial cluster suggests remarkable range dynamics in *P. baton* in the past glacial and interglacial phases, with most of their results being overwritten later on, but with geographically small rear edge remnants, hereby supporting glacial refugia in southern France^78^ and Corsica^65^.

In several cases, *Wolbachia* infection was observed to match mitochondrial phylogenies in butterflies, but the nuclear information coincided with life history traits (^79^ for *Eurema hecabe* (Linnaeus, 1758), Pieridae^38^ for *Hypolimnas* Hübner, 1819 spp., Nymphalidae). In these examples, *Wolbachia* is suspected to mediate interspecific introgression. In our case of *Pseudophilotes*, however, the hybridization probably happened regardless of *Wolbachia. Wolbachia* presence (in Croatian samples) or absence (in French, Italian, and Corsican *P. baton*) rather provides additional evidence for hybridization.

### Limitations and future directions

We addressed the patterns and diversity in *Wolbachia* infection across a broad geographic range in several species. However, the exact mechanisms whether and how *Wolbachia* affects these butterfly hosts remains undiscovered. Routes to discovering them may include breeding experiments and antibiotic treatments of infected populations. Such strategy would identify cytoplasmic incompatibility, male killing or feminisation (e.g.^80^). Males of the butterflies inspected are not notably rare in natural conditions and our dataset contained males bearing all *Wolbachia* alleles detected here (Supplementary Dataset S1). To identify the exact *Wolbachia* strains, the whole MLST typing^39^ for each *wsp* allele would be necessary. As the two used *Wolbachia* markers originate from different parts of *Wolbachia* genome and the information, which they deliver, is congruent, we believe that this additional information would not change our conclusions, but rather refine them.

The exact distribution of *Aricia artaxerxes* in temperate Europe is still obscured, and all putative populations should be examined using DNA barcoding. Using high-throughput sequencing of samples from the Eastern Alps, where *A. agestis* and *A. artaxerxes* barcodes coexist at the same site, would distinguish between past hybridization and horizontal transfer of *Wolbachia*.

### Synthesis

Taken together, in the two analysed Polyommatinae groups, we observed several different relations between *Wolbachia* infection and mitochondrial DNA of their hosts. First, presence of *Wolbachia* caused impoverishment in mitochondrial haplotypes, when compared to uninfected relatives. Second, within a species (or species complex), different strains of *Wolbachia* usually infect different mitochondrial groups (clusters). Third, a possible horizontal transfer of *Wolbachia* occurred between sympatric populations. Fourth, *Wolbachia* presence, absence or allele identity served as an additional marker of hybridization in a contact zone.

Specifically, in the case of *Aricia*, the two taxa with diverging mitochondrial structures mirroring *Wolbachia* prevalence were probably more spatially separated in the past, and both a hybridization^60^ and a horizontal transmission of *Wolbachia* likely occurred during Holocene in the contact areas of both species. In the case of *Pseudophilotes*, we observed several poorly defined taxa, with different mitochondrial clusters infected by different *Wolbachia* strains, or remaining uninfected by the bacteria. Alongside the contact zone, *Pseudophilotes* species hybridized, which was revealed by the discrepancy between the morphological trait (genital structure) and the mitochondrial signal corresponding to *Wolbachia* infection.

Palaearctic Polyommatinae butterflies were exposed to the late Cenozoic cooling of the Earth, habitat opening and climate alternations. Their host plants are often herbs occurring in grasslands, chemically protected from ungulate grazing. Many taxa rapidly diversified under such conditions. The population divergence leading to speciation is often not completed, and we observe different levels of its completeness in various taxa^81^. *Aricia* spp. and *Pseudophilotes* spp. represent two different levels of such diversification. Species-rich radiations seem to be common in other Polyommatinae (e.g. the *Polyommatus coridon* group^82,83^ and *Agrodiaetus* species with chromosomal fissions^84^). *Wolbachia* and other symbiotic bacteria are suspected drivers of arthropod evolution^85^. The species might even lose the infection after some time^86^, so that we could not reveal the culprit of diversification. We suggest that *Wolbachia* infection plays an important role in the mitochondrial diversity of butterflies and this should be taken into account in all phylogeographic and cryptic diversity studies involving mitochondrial DNA. *Wolbachia* infection may prevent inferring the host species’ mitochondrial history, but knowledge on the distribution of *Wolbachia* alleles can be helpful in understanding the mitochondrial patterns and biogeographic scenarios.

## Supplementary Information

Supplementary Dataset S1.

Supplementary Figures.

## Data Availability

All sequences obtained in this study are stored in GenBank (Accession numbers: butterfly COI *Aricia agestis*/*A. artaxerxes*: MT883793–MT883959; COI *Pseudophilotes baton* species complex: MT878244–MT878363; *Wolbachia* wsp from *A. agestis* and *A. artaxerxes:* MT950442–MT950530, and coxA: MT950363–MT950441; wsp from *P. baton* species complex: MT890972–MT891001, and coxA: MT891002–MT891029). The codes of each specimen are in Supplementary Dataset S1. The Nexus alignments are stored at the Figshare repository https://doi.org/10.6084/m9.figshare.12917426.v1.
