# Supplementary material for: Wolbachia affects mitochondrial population structure in two systems of closely related Palaearctic blue butterflies
Source: Sci Rep. 2021 Feb 4;11:3019. doi: 10.1038/s41598-021-82433-8 (PMC7862691; doi:10.1038/s41598-021-82433-8)
Supplement: Supplementary file 2 — Supplementary Figures. [file 41598_2021_82433_MOESM2_ESM.docx]

***Wolbachia* affects mitochondrial population structure in two systems of closely related Palaearctic blue butterflies**

Alena Sucháčková Bartoňová^1 *^, Martin Konvička^1, 2^, Jana Marešová^1, 2^, Martin Wiemers^3^, Nikolai Ignatev^1, 2^, Niklas Wahlberg^4^, Thomas Schmitt^3, 5^, Zdeněk Faltýnek Fric^1^

1 Institute of Entomology, Biology Centre CAS, Branisovska 31, 37005 Ceske Budejovice, Czech Republic

2 Faculty of Science, University of South Bohemia, Branisovska 1760, 37005 Ceske Budejovice, Czech Republic

3 Senckenberg Deutsches Entomologisches Institut, Eberswalder Str. 90, 15374 Müncheberg, Germany

4 Lund University, Department of Biology, Sölvegatan 37, Lund, Sweden

5 Zoology, Institute of Biology, Faculty of Natural Sciences I, Martin Luther University Halle-Wittenberg, 06099 Halle (Saale), Germany

* Corresponding author

**Scientific Reports 2020**

**SUPPLEMENTARY FIGURES**

**Supplementary Figure S1.** a) Bayesian tree (Beast) of haplotypes of *Aricia* butterflies. b–e) Bayesian trees (MrBayes) of *Wolbachia* b) wsp sequences from *Aricia agestis* and *A. artaxerxes*, c) coxA sequences from *Aricia agestis* and *A. artaxerxes*, d) wsp sequences from *Pseudophilotes* *baton* species complex, and e) coxA sequences from *Pseudophilotes baton* species complex. Branch labels show posterior probabilities. The tree was visualized in FigTree v. 1.3.1 (https://github.com/rambaut/figtree). Graphics was compiled in Graphic for Mac v. 3.1 (https://www.graphic.com/mac).


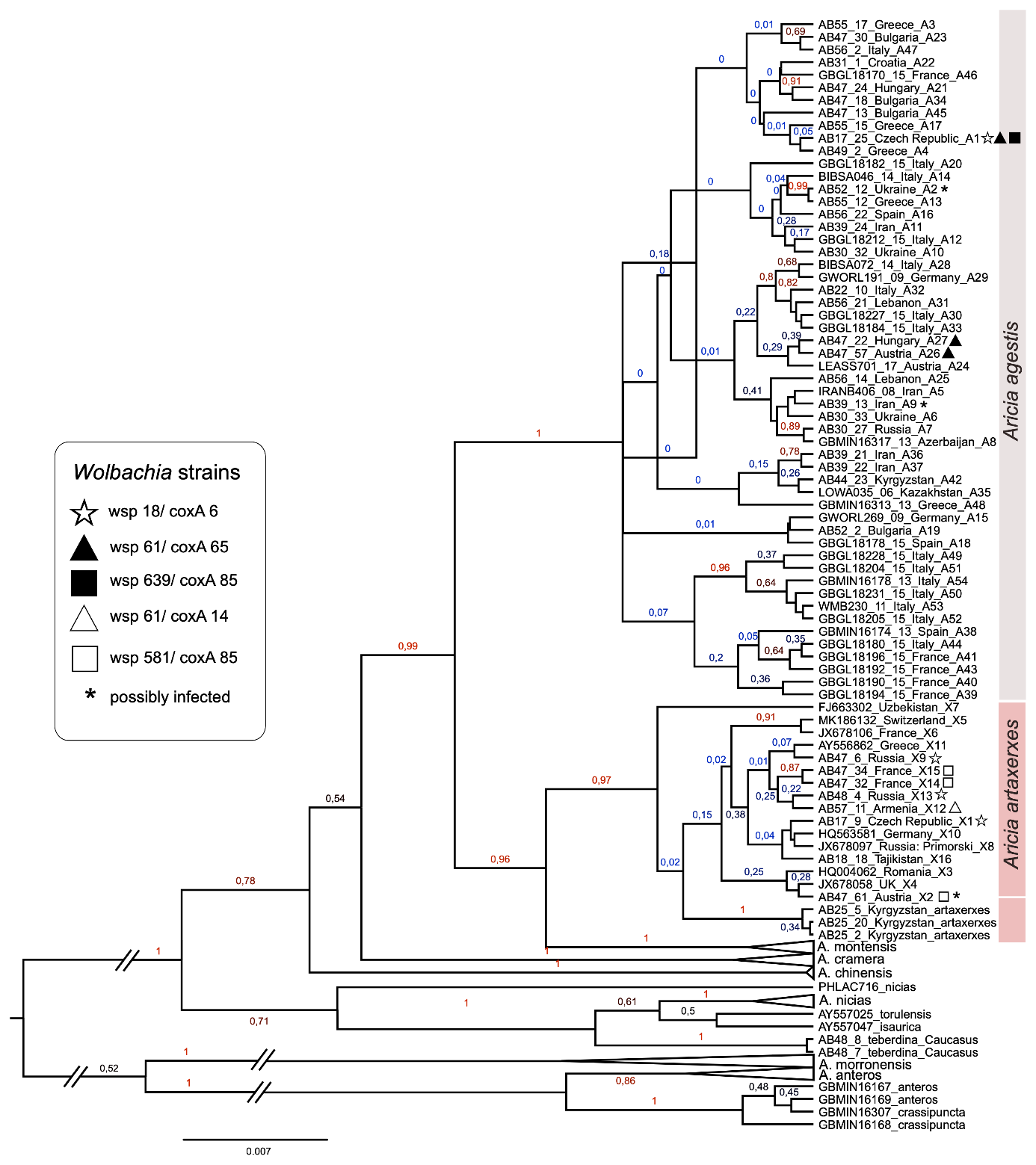


**Figure S1 a.** *Aricia* butterflies.

**
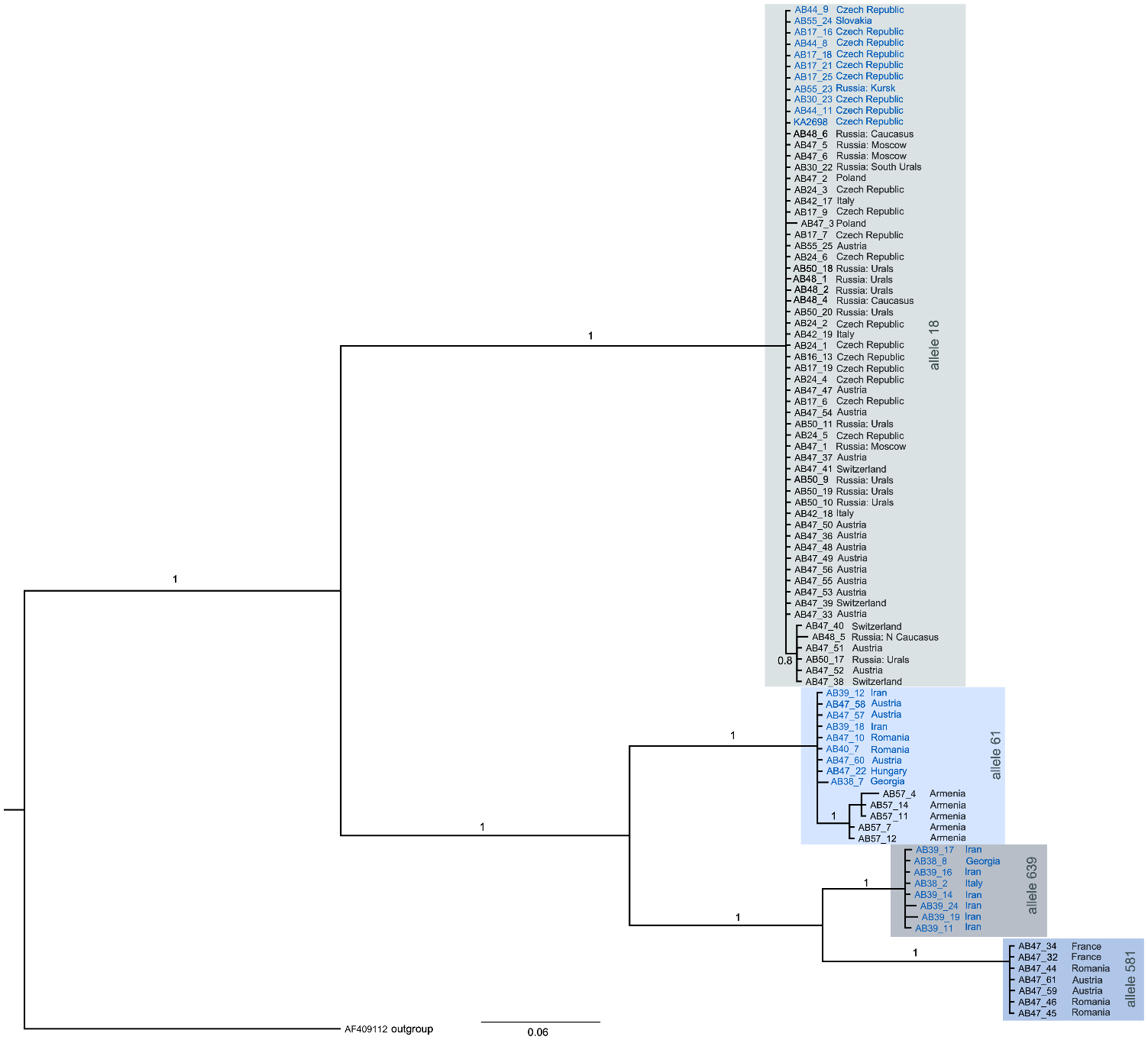
**

**Figure S1 b.** *Wolbachia* wsp from *Aricia agestis* (blue letters) and *A. artaxerxes* (black letters).

**
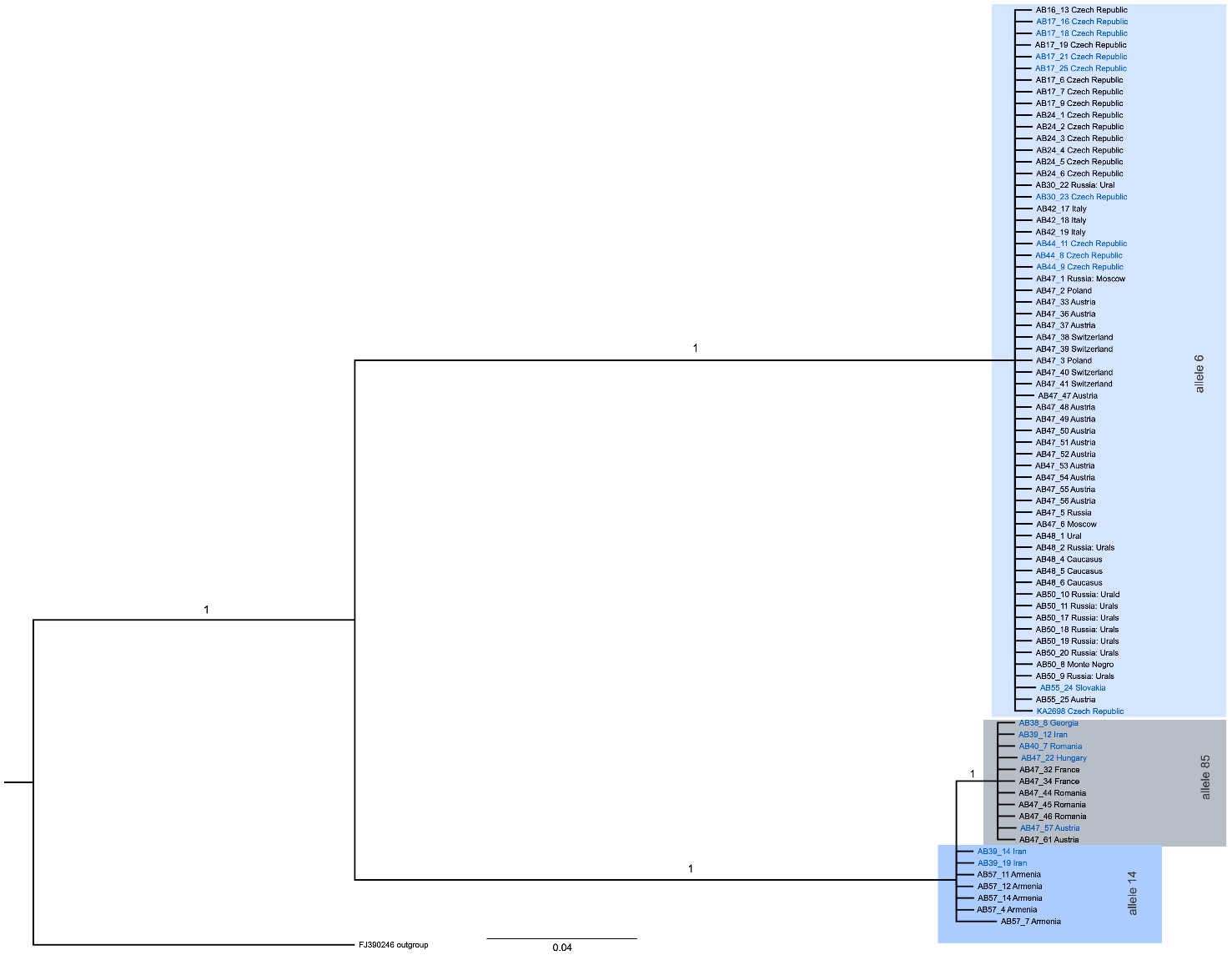
**

**Figure S1 c.** *Wolbachia* coxA from *Aricia agestis* (blue letters) and *A. artaxerxes* (black letters)*.*

**
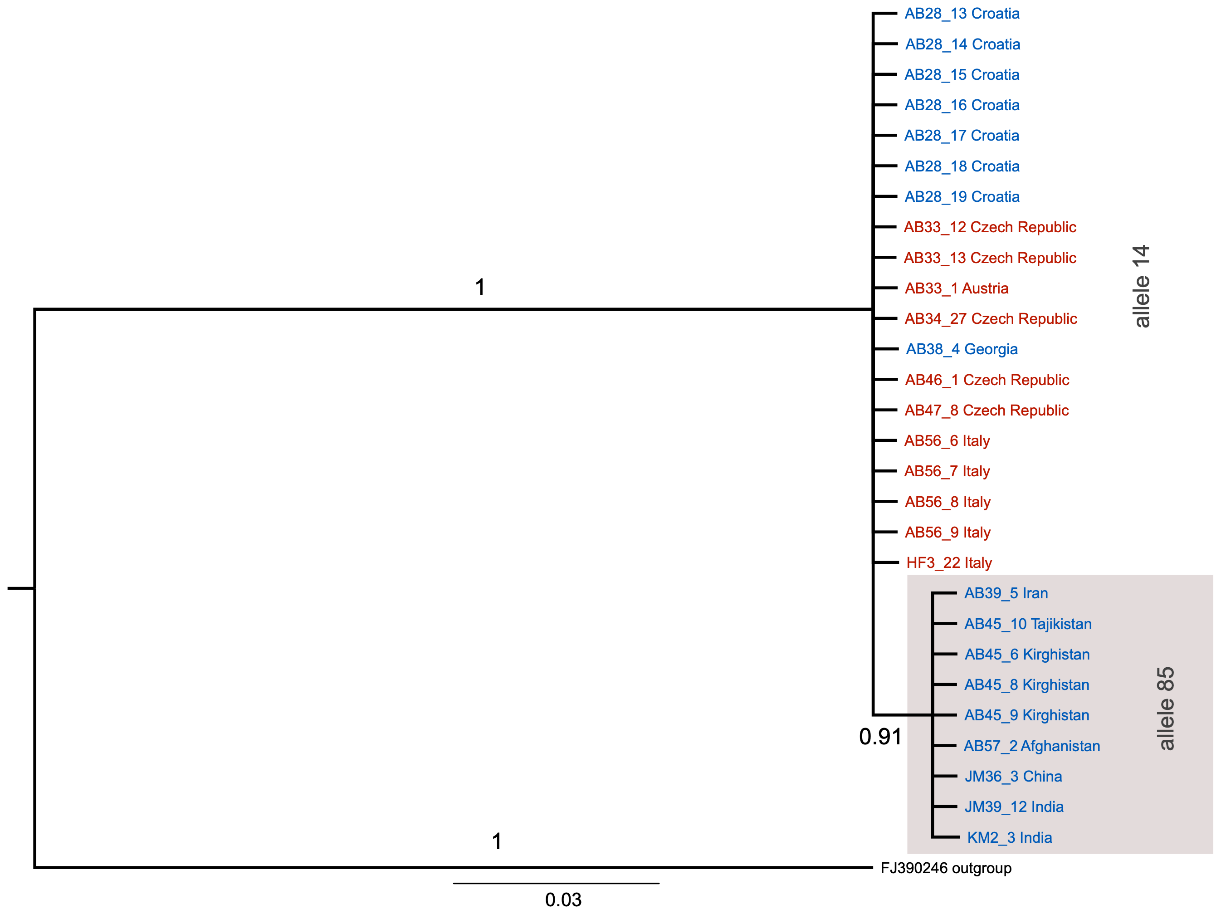
**

**Figure S1 d.** *Wolbachia* wsp from *Pseudophilotes* *baton* species complex (blue letters: *P. vicrama*, red letters: *P. baton*).

**
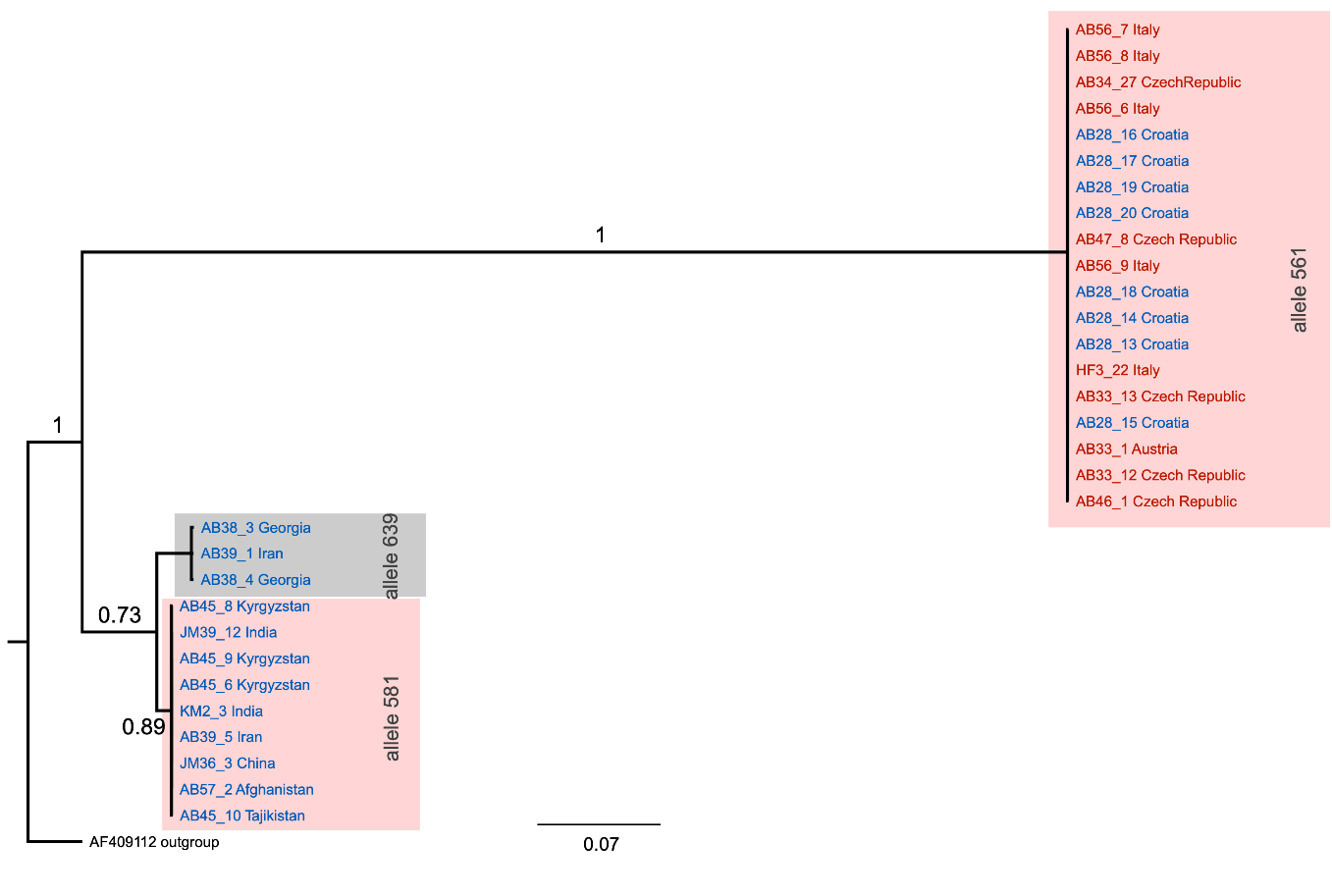
**

**Figure S1 e.** *Wolbachia* coxA from *Pseudophilotes baton* species complex (blue letters: *P. vicrama*, red letters: *P. baton*).


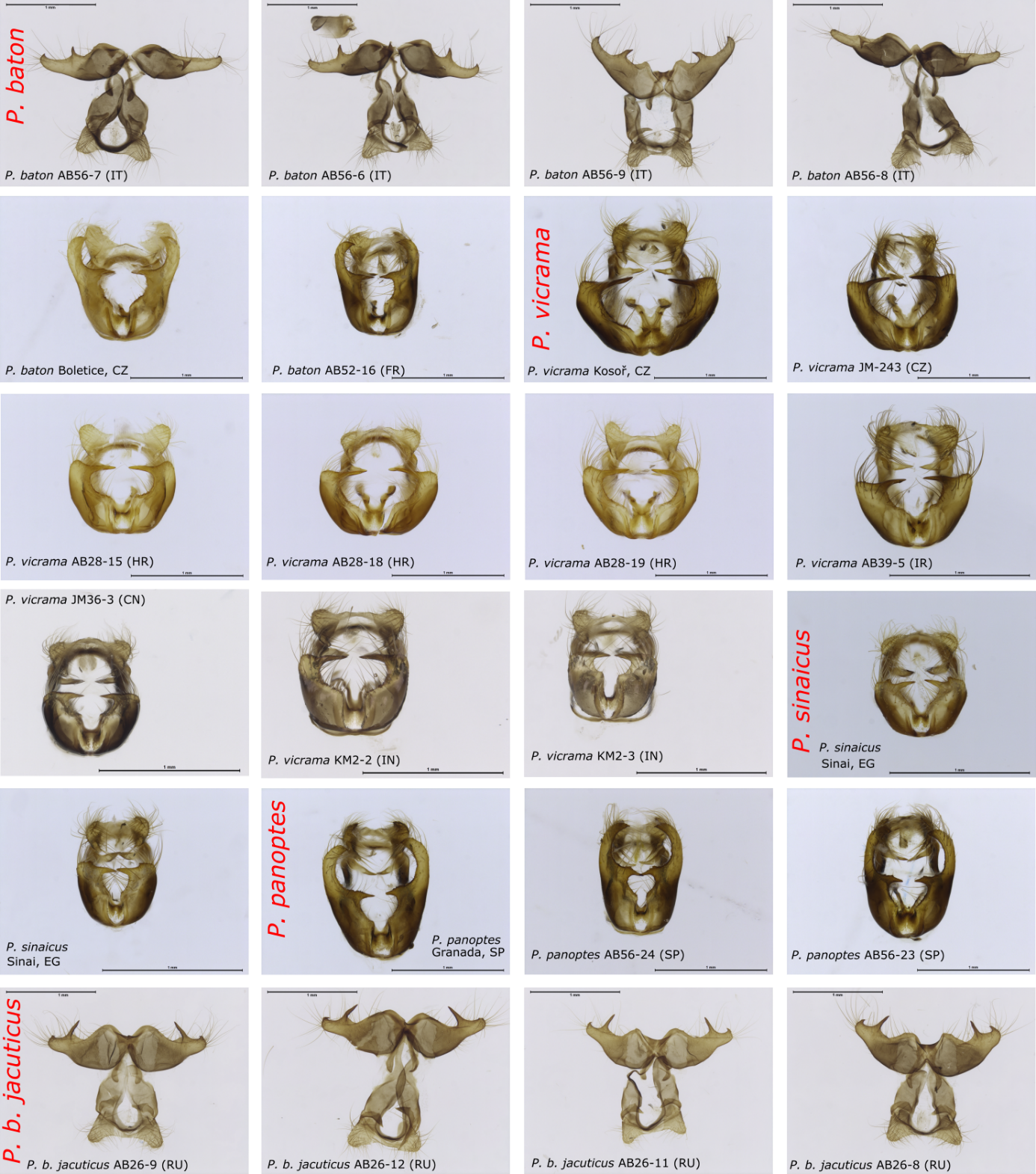


**Supplementary Figure S2.** The diversity of the male genital valvae in *Pseudophilotes baton* species complex. Samples from the Croatian *P. vicrama* population (positive for *Wolbachia*, host barcode: BAPSp2 found in *P. baton*) have one tooth on valva, a type found in *P. vicrama*. A sample from the French *P. baton* population (negative for *Wolbachia*, host barcode: BAPSp1 found mostly in *P. vicrama*, but unique haplotypes for the population) has two teeth on valva, a form typical for *P. baton*. Graphics was compiled in Inkscape v. 1.0 (https://inkscape.org/).
